# Molecular detection of Shiga toxin-producing *Escherichia coli* in wild rats from urban areas of Banyuwangi District, Indonesia: Implications for zoonotic risk and public health

**DOI:** 10.14202/vetworld.2025.3109-3119

**Published:** 2025-10-20

**Authors:** Ratih Novita Praja, Elham Zahrudin, Ryanka Edila, Aditya Yudhana, April Hari Wardhana, Dyah Haryuningtyas Sawitri, Tati Ariyanti, Faidah Rachmawati, Alfian Dzaka Fadhil Ramadhan, Frenky Laksana Putra, Muhammad Aqil Kurnianto, Aldi Gusnizar Rizaldy Tanjung, Priyono Priyono, Ristiyanto Ristiyanto, Makoto Matsubayashi

**Affiliations:** 1 Veterinary Medicine Study Program, Department of Health and Life Sciences, Faculty of Health, Medicine, and Life Sciences, Universitas Airlangga, Banyuwangi, Indonesia; 2 Research Group for Animal Biomedical and Conservation, Universitas Airlangga, Surabaya, Indonesia; 3 Doctoral Program of Veterinary Science, Faculty of Veterinary Medicine, Universitas Airlangga, Surabaya, Indonesia; 4 Research Center for Veterinary Science, Research Organization for Health, National Research and Innovation Agency (BRIN), Cibinong, Bogor, Indonesia; 5 Research Center for Behavioral and Circular Economics, Research Organization of Governance, Economy, and Community Welfare, National Research and Innovation Agency (BRIN), Jakarta, Indonesia; 6 Research Center for Public Health and Nutrition, Organization Research for Health, National Research and Innovation Agency (BRIN), Cibinong, Bogor, Indonesia; 7 Laboratory of Veterinary Immunology, Department of Bioenvironmental Science, Graduate School of Veterinary Science, Osaka Metropolitan University, Osaka, Japan

**Keywords:** Banyuwangi, *Escherichia coli*, infectious disease, one health, public health, Shiga toxin, wild rats, zoonosis

## Abstract

**Background and Aim:**

*Escherichia coli* strains producing Shiga toxins (*stx1*, *stx2*) are important zoonotic pathogens. Wild rats, common in urban environments with poor sanitation, may act as reservoirs and contribute to environmental contamination. This study aimed to detect Shiga toxin-producing *E. coli* (STEC) in wild rats captured from slum-adjacent urban areas of Banyuwangi District, Indonesia.

**Materials and Methods:**

From August to October 2024, a total of 100 wild rats (32% *Rattus norvegicus*, 68% *Rattus tanezumi*) were trapped in Kampung Mandar and Lateng Villages. Rectal swabs were collected and cultured on eosin methylene blue agar, followed by biochemical and sugar fermentation tests for preliminary identification. Molecular confirmation of *E. coli* was performed using a polymerase chain reaction (PCR) targeting *cydA* and *lacY*. Confirmed isolates were further screened for *stx1* and *stx2* genes. Statistical analysis was performed using Chi-square tests.

**Results:**

PCR confirmed *E. coli* in 57% (57/100) of rats. Among these, 47.36% (27/57) carried the *stx1* gene, 7.01% (4/57) carried the *stx2* gene, and 3.51% (2/57) harbored both genes. The prevalence of *E. coli* was significantly higher in *R. tanezumi* than in *R. norvegicus* (p = 0.000), but toxin gene distribution showed no significant differences across species, sex, or locations.

**Conclusion:**

This study provides the first molecular evidence of wild rats in Banyuwangi carrying *E. coli* strains harboring *stx1* and *stx2* genes. The findings highlight the role of urban rodents as reservoirs of zoonotic STEC and underline the need for routine monitoring, improved waste management, and integrated One Health strategies to mitigate zoonotic transmission risks in high-density urban environments.

## INTRODUCTION

Rats, as synanthropic small mammals inhabiting residential areas, represent a considerable public health risk. They act as reservoirs for numerous pathogens, including zoonotic *Escherichia coli* [1, 2]. While most *E. coli* strains are commensal and nonpathogenic, certain strains harbor virulence factors that can cause diseases such as gastroenteritis, urinary tract infections, and bacteremia [3, 4]. Some pathogenic variants may also compromise the immune system by inducing lymphocyte depletion in lymphoid tissues [5]. Based on their virulence mechanisms, pathogenic *E. coli* are grouped into seven pathotypes: enteropathogenic *E. coli* (EPEC), enterotoxigenic *E. coli*, enterohemorrhagic *E. coli* (EHEC), enteroaggregative *E. coli*, enteroinvasive *E. coli*, diffusely adherent *E. coli*, and adherent-invasive *E. coli* strains [6]. Among these, EHEC, particularly serotype O157:H7, is the most virulent, producing Shiga toxins encoded by the *stx1* and *stx2* genes [7]. These toxins, also known as verotoxins, exert cytotoxic effects on Vero cells and are linked to severe human illnesses, including hemorrhagic colitis and hemolytic uremic syndrome (HUS) [8].

The two major Shiga toxins differ in pathogenic potential. Although *stx1* is generally associated with milder symptoms, it can still contribute to HUS [9, 10]. It has also been shown to stimulate tumor necrosis factor-alpha release, potentially increasing the susceptibility of brain endothelial cells to toxin-mediated injury [11]. In contrast, *stx2* is estimated to be up to 1,000 times more virulent, owing to its stronger receptor affinity and greater capacity to induce severe cellular damage [12]. These differences highlight the critical need to detect both toxin genes in potential animal reservoirs for effective public health surveillance.

In Indonesia, investigations into Shiga toxin-producing *E. coli* (STEC) in wild rodents are scarce, especially in urban environments. Wardhana *et al*. [13] reported that Kampung Mandar and Lateng Villages in Banyuwangi District, formerly categorized as slums, remain adjacent to the highly unsanitary Cemara slum area, particularly Karangrejo Village. These locations are characterized by poor sanitation and a high prevalence of parasitic infections, creating a favorable environment for the transmission of rodent-borne pathogens.

Although STEC is recognized as an important zoonotic pathogen worldwide, studies on its occurrence in wild rodents in Indonesia remain limited. Most existing research in the country has focused on livestock, food products, or human cases, with only a few reports investigating rodents as potential reservoirs. International studies have highlighted the role of urban rats in harboring pathogenic *E. coli* strains, yet comparable data from Southeast Asia, particularly from Indonesian urban environments, are scarce. Moreover, the majority of available studies rely on conventional culture or biochemical methods, which may underestimate true prevalence compared to molecular detection. Importantly, no published studies to date have documented the simultaneous presence of *stx1* and *stx2* genes in wild rats from densely populated Indonesian urban settings. This lack of information represents a significant gap in understanding the zoonotic risks posed by synanthropic rodent populations living in proximity to humans, especially in areas with poor sanitation and high human–rat interaction.

The present study was designed to address this knowledge gap by conducting molecular detection of STEC in wild rats captured from urban areas of Banyuwangi District, Indonesia. Specifically, the study aimed to: (i) isolate and confirm *E. coli* from rectal swabs of wild rats using culture, biochemical, and polymerase chain reaction (PCR) methods; (ii) detect the presence of *stx1* and *stx2* genes to determine the prevalence of toxin-producing strains; and (iii) analyze associations between rat species, sex, location, and infection status. By providing the first molecular evidence of *stx1* and *stx2*-positive *E. coli* in urban wild rats in Banyuwangi, this research contributes to a better understanding of rodent-borne zoonotic threats in Indonesia and underscores the importance of integrated One Health surveillance strategies for high-risk urban environments.

## MATERIALS AND METHODS

### Ethical approval

The Ethical Clearance Committee of the Faculty of Veterinary Medicine, Universitas Gadjah Mada, Yogyakarta, Indonesia, reviewed and approved this study under certificate No. 10/EC-FKH/int./2024.

### Study period and location

The study was conducted from August to October 2024. A total of 100 wild rats were captured in the villages of Kampung Mandar (8.2091°S, 114.3832°E) and Lateng (8.1994°S, 114.3772°E). These villages are densely populated and located adjacent to the Cemara slum area, particularly Karangrejo Village (SK No. 188/67/Kep/429.011/2023).

### Species identification

Single live traps were deployed each evening at 7:00 p.m. and retrieved at 5:00 a.m. the following morning. A total of 10 traps were set in each village per night, with a total of 20 trapping nights conducted across the two villages. Salted fish and baked coconut were used as bait in the traps. The average trap success rate was 50%. The captured rats were transported in sealed containers to the laboratory for identification and further processing. Before sample collection, the animals were anesthetized through intramuscular injection of ketamine (50 mg/kg body weight [BW]) and xylazine (5 mg/kg BW).

Species identification was carried out by measuring external morphological features in a prone position using a ruler, while BW was recorded with a digital scale. The measured parameters included total body length, tail length, hind foot length, and ear length. These measurements were used to differentiate species according to standard morphological identification keys [13]. Rodents were identified based on their external morphological characteristics, including sex determination and anatomical measurements. The BW of euthanized individuals was measured using a digital precision balance. External morphometric parameters were recorded using a Vernier caliper, covering five specific traits: head and body length, tail length, hind-foot length, ear length, and skull length. Morphometric data were then compared with standard taxonomic references for identification [14].

### Bacterial isolation and phenotypic identification

Following the administration of injectable ketamine anesthesia, *E. coli* was isolated from wild rats by rectal swab testing using a sterile cotton swab inserted 4–6 cm into the rectum. The swab was then immersed in a test tube containing physiological NaCl solution to maintain moisture. The rectal swab was streaked onto eosin methylene blue agar (EMBA) (HiMedia, Mumbai, India) and incubated at 37°C for 18–24 h under aerobic conditions. The suspected *E. coli* colonies displayed a metallic green sheen, a lustrous appearance, and a round shape with smooth, flat edges. Isolated colonies were subsequently subcultured onto fresh EMBA medium for further identification. Presumptive *E. coli* colonies were then subjected to Gram staining (Merck, Darmstadt, Germany). The isolates were confirmed to be *E. coli* by the microscopic observation of short red bacilli.

### Biochemical and fermentation of sugar

*E. coli* was identified using IMViC biochemical tests, consisting of the Indole test (Merck), Methyl Red (MR) test (HiMedia), Voges-Proskauer (VP) test (HiMedia), and Simmons Citrate test (HiMedia). The specimen was identified as *E. coli*, confirmed by positive Indole results indicated by a red ring after the addition of Kovac’s reagent, and positive MR results confirmed by the red color after the addition of MR indicator. The VP test was confirmed to be negative by the absence of color change after the addition of Barritt’s reagents A and B, and the Simmons Citrate test was confirmed to be negative by the absence of color change from green to blue.

Carbohydrate fermentation tests were performed by inoculating bacterial colonies onto media containing glucose, sucrose, lactose, maltose, and mannitol (all from HiMedia), followed by incubation at 37°C for 18–24 h. The fermentation test for *E. coli* yielded positive results, indicated by a color change of the medium from red to yellow and gas production in the Durham tube (HiMedia).

### DNA extraction and PCR of *E. coli*

To obtain pure isolates, two to three presumptive *E. coli* colonies grown on EMBA were subcultured onto nutrient agar plates. The cultivated colonies were harvested using a sterile inoculating loop and suspended in sterile distilled water to achieve a final concentration of 1 × 10^7^ colony-forming units/mL. A 1 mL aliquot of the bacterial suspension was transferred into a sterile microcentrifuge tube and centrifuged at 300 × *g* for 5 min to form a pellet. DNA extraction was then performed using the resuspended pellet according to the manufacturer’s protocol for genomic DNA isolation from cultured cells (Geneaid, Taiwan) [15].

### Detection of the *stx1* and *stx2* genes

PCR analysis was used to confirm the identification of *E. coli* based on biochemical and sugar tests, using universal primers targeting *cydA* (encoding cytochrome bd-I ubiquinol oxidase subunit 1) and *lacY* (encoding lactose permease). Additional PCR assays were performed to detect toxin genes using primers specific for *stx1* and *stx2*. Negative controls (*E. coli* ATCC strains lacking *stx* genes) and positive controls were included in all PCR reactions to prevent contamination and ensure specificity. Table 1 [14, 15] lists the nucleotide sequences of each primer.

**Table 1:** List of primers used in the present study.

No.	Primers	Sequence 5’- 3’	Products (bp)	Reference
1	*cydA-*F	CGTATGGAGATGGTGAG	515	[14]
	*cydA-*R	GTAGAACCAGAACGCAGT		
2	*lacY*-F	TTCCCACCGATGCGATT	192	[14]
	*lacY*-R	GTCACTGTATGTTATTGGCG		
3	*st×1*-F	TGTAACTGGAAAGGTGGAGTATACA	210	[15]
	*st×1*-R	GCTATTCTGAGTCAACGAAAAATAAC		
4	*st×2*-F	GTTTTTCTTCGGTATCCTATTCC	484	[15]
	*st×2*-R	GATGCATCTCTGGTCATTGTATTAC		

*R. norvegicus* = R*attus norvegicus*, *R. tanezumi* = R*attus tanezumi*, F = Forward, R = Reverse

Each PCR reaction contained 25 μL of reaction mixture, including MyTaq HS red mix (Bioline, Meridian Bioscience, UK), primers, template DNA, and nuclease-free water. All PCR runs included a positive control with verified *E. coli* DNA containing the target gene and a negative control using nuclease-free water instead of template DNA. These controls validated the specificity and reliability of amplification and excluded the possibility of contamination or false positives.

The PCR conditions for the universal primers (*cydA* and *lacY*) were as follows: initial denaturation at 95°C for 1 min (1 cycle); denaturation at 95°C for 15 s (35 cycles); annealing at 53°C for 15 s (35 cycles); extension at 72°C for 15 s (35 cycles); and final extension at 72°C for 10 min (1 cycle). For *stx1* and *stx2* primers, the conditions were similar, except the annealing temperature was set at 50°C for 15 s over 35 cycles. PCR products were visualized on a 1.5% Tris-acetate-Ethylenediaminetetraacetic acid agarose gel with a 1,000-bp DNA ladder. The gels were stained with FluoroSafe Gel Stain (1^st^ Base, Singapore), electrophoresed at 100 V for 30 min, and visualized under an ultraviolet transilluminator (Analytik Jena, Jena, Germany).

### Statistical analysis

All data were entered into Microsoft Excel 2019 (Microsoft Corp., Washington, USA) and analyzed using the Chi-square test in Statistical Package for the Social Sciences version 23 (IBM Corp., NY, USA), with a 95% confidence level. Chi-square tests were performed to evaluate associations between categorical variables, including sampling location (Kampung Mandar vs. Lateng), rat species (*Rattus norvegicus* vs. *Rattus tanezumi*), sex (male vs. female), and infection status with *E. coli* and its toxin genes (*stx1*, *stx2*). These variables were analyzed to assess distribution differences and potential relationships with pathogen prevalence.

## RESULTS

### Rat capture and distribution

A total of 100 wild rats were captured during the study, comprising 32 from Kampung Mandar Village and 68 from Lateng Village. Statistical analysis revealed a significant difference in the number of wild rats captured between the two villages (p < 0.05; p = 0.000).

### Species composition and sex ratio

Two rat species were identified: *R. norvegicus* (32%) and *R. tanezumi* (68%). Although *R. tanezumi* was more prevalent, the difference in species distribution was not statistically significant (p = 0.569). The overall sex ratio was nearly equal, with 51 males and 49 females (p = 0.771).

### Detection of *E. coli*

**Figure 1:**
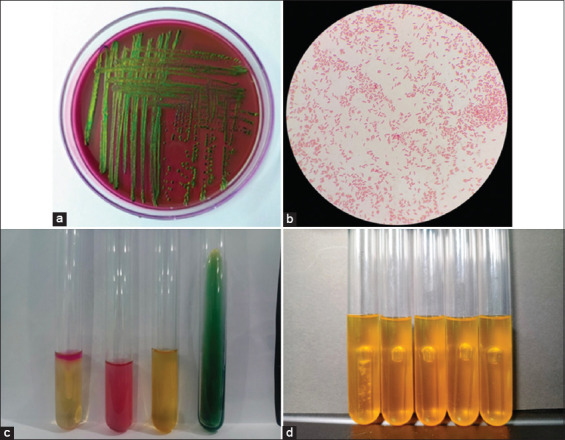
Isolation and identification of *Escherichia coli* isolated from the rectal swab of rats. (a) *E. coli* colonies grown on eosin methylene blue agar medium appear metallic green with black dots. (b) Gram-stained bacteria show coccobacilli shape and red coloration. (c) Indole, Methyl Red, Voges-Proskauer, and Citrate test results from left to right: Indole (+) showed a red ring; Methyl Red (+) changed from yellow to red with Methyl Red reagent; Voges-Proskauer (−) showed no color change with alpha-naphthol 5% and potassium hydroxide (KOH) 40%; and Simmons Citrate Agar (SCA) (−) showed no color change. (d) Sugar fermentation test results: glucose (+), sucrose (+), lactose (+), maltose (+), and mannitol (+), indicated by a color change from red to yellow.

Initial screening on EMBA medium indicated the presence of *E. coli* in 79% of rats, which was further confirmed by biochemical and sugar tests (Figures 1a–d). Subsequent PCR verification using the *cydA* and *lacY* genes confirmed 57 of 69 isolates (82.6%, 95% confidence interval [CI]: 73.6%–91.6%) as true *E. coli* (Table 2). The majority of confirmed *E. coli*-positive rats were from Lateng Village (68.42%) compared with Kampung Mandar (31.57%), a difference that was statistically significant (p = 0.008).

**Table 2:** Results of *E. coli* identification based on biochemical and sugar tests, including PCR analysis results utilizing universal primers, *cydA*, and *lacY*.

No	Locations	Results	Biochemical and sugars tests				Total	
	
			*R. norvegicus*		*R. tanezumi*			
		
			Male	Female	Male	Female	Male	Female
1	Kampung Mandar	Positive	1	4	7	7	8	11
		Negative	3	1	6	3	9	4
2	Lateng	Positive	6	10	20	14	26	24
		Negative	3	4	5	6	8	10
Total		Positive	7	14	27	21	34	35
		Negative	6	5	11	9	17	14
		Results	Molecular analyses using universal primers (*cydA* and *lacY*)					
1	Kampung Mandar	Positive	1	3	7	7	8	10
		Negative	0	1	0	0	0	1
2	Lateng	Positive	5	5	19	10	24	15
		Negative	1	5	1	4	2	9
Total		Positive	6	8	27	21	32	25
		Negative	1	6	11	9	2	10

*R. norvegicus* = *Rattus norvegicus*, *R. tanezumi* = *Rattus tanezumi*, *Escherichia coli* = *Escherichia coli*, PCR = Polymerase chain reaction

### *E. coli* infection rates by species and sex

The infection rates of *E. coli* were significantly higher in *R. tanezumi* (75.44%) compared with *R. norvegicus* (24.56%; p = 0.000). No significant differences were observed between sexes, with infection rates of 56.14% in males and 43.86% in females (p = 0.423).

### Prevalence of Shiga toxin genes

Among *E. coli*-positive rats, 47.36% (27/57) harbored the *stx1* gene (95% CI: 34.4%–60.3%). The prevalence of *stx1* was consistent across villages (Kampung Mandar, 50%; Lateng, 46.15%; p = 0.787), species (*R. norvegicus*, 50%; *R. tanezumi*, 46.51%; p = 0.820), and sex (males, 46.87%; females, 48%; p = 0.933) (Tables 3 and 4).

**Table 3:** Results of molecular detection of *st×1* and *st×2* from *E. coli* isolated from rats captured in two villages of Banyuwangi Districts.

No	Locations	Results	PCR analyses for *st×1*				Total	
	
			*R. norvegicus*		*R. tanezumi*	
		
			Male	Female	Male	Female	Male	Female
1	Kampung Mandar	Positive	0	1	4	4	4	5
		Negative	1	2	3	3	4	5
2	Lateng	Positive	4	2	7	5	11	7
		Negative	1	3	12	5	13	8
Total		Positive	4	3	11	9	15	12
		Negative	2	5	15	8	17	13
		Results	PCR analyses for *st×2*					
1	Kampung Mandar	Positive	0	0	0	0	0	0
		Negative	1	3	7	7	8	10
2	Lateng	Positive	1	0	2	1	3	1
		Negative	4	5	17	9	21	4
Total		Positive	1	0	2	1	3	1
		Negative	5	8	24	16	29	24

*R. norvegicus* = *Rattus norvegicus*, *R. tanezumi* = *Rattus tanezumi*, *Escherichia coli* = *Escherichia coli*, PCR = Polymerase chain reaction

**Table 4:** Detection results of single and double toxins (*st×1* and *st×2*) in *E. coli* isolated from rectal swab of rats based on PCR analysis.

No.	Species	Parameter	Location		Total

			Kampung Mandar	Lateng	
1.	*R. norvegicus*	RPE	4	10	14
		RNE	5	13	18
		*st×1* (+)	1	6	7
		*st×2* (+)	0	1	1
		*st×1* (+); *st×2* (+)	0	1	1
		*st×1* (−); *st×2* (−)	3	4	7
2.	*R. tanezumi*	RPE	14	29	43
		RNE	9	16	25
		*st×1* (+)	8	12	20
		*st×2* (+)	0	3	3
		*st×1* (+); *st×2* (+)	0	1	1
		*st×1* (−); *st×2* (−)	6	15	21
Total		RPE	18	39	57
		RNE	14	29	43
		*st×1* (+)	9	18	27
		*st×2* (+)	0	4	4
		*st×1* (+); *st×2* (+)	0	2	2
		*st×1* (−); *st×2* (−)	9	19	28

RPE = Rats with positive *E. coli*, RNE = Rats with negative *E. coli*, *st×1* (+) = *E. coli* detected by PCR with positive *st×1* gene, *st×2* (+) = *E. coli* detected by PCR with positive *st×2* gene, *st×1* (−) = *E. coli* detected by PCR with negative *st×1* gene, *st×2* (−) = *E. coli* detected by PCR with negative *st×2* gene, *R. norvegicus* = *Rattus norvegicus*, *R. tanezumi* = *Rattus tanezumi*, *E. coli = Escherichia coli*, PCR = Polymerase chain reaction

Only 4 rats (7.01%) carried the *stx2* gene (95% CI: 0.4%–13.6%), all of which originated from Lateng Village. None were detected in Kampung Mandar. A slightly higher prevalence was observed in *R. tanezumi* (6.97%) compared with *R. norvegicus* (7.14%), though the difference was not significant (p = 0.983). Similarly, there was no significant difference between males (9.37%) and females (4%; p = 0.431).

### Dual-toxin-producing isolates

Isolates harboring both *stx1* and *stx2* genes were rare, detected in only 2 cases (2.94%, 95% CI: 0%–8.3%). Both were identified in Lateng Village, one from each of the two rat species.

### Summary of findings

Overall, these results demonstrate that wild rats in Kampung Mandar and Lateng Villages act as reservoirs of pathogenic *E. coli*. The presence of both *stx1* and *stx2* genes underscores the potential zoonotic risk associated with urban rodent populations.

## DISCUSSION

### Species composition of wild rats

This study identified two wild rat species in the urban coastal settings of Banyuwangi: *R. tanezumi* (68%) and *R. norvegicus* (32%). The dominance of *R. tanezumi* aligns with rodent surveys from Bogor [16] and Banyuwangi [17], where the species is well adapted to tropical urban environments. By contrast, *R. norvegicus* remains the dominant species in cities such as Surabaya [18], indicating that habitat type, human population density, and food availability have a strong influence on regional variation. Due to inconsistent fecal availability in wild rats, rectal swabs, shown to allow faster detection of *stx2*-positive *E. coli* [19], were used, with PCR confirming 57% positivity versus 69% by biochemical methods, reflecting PCR’s higher specificity, in line with previous reports of *E. coli* in dairy environments [20], raw milk [21], and contaminated beef, including O157:H7 strains [22].

### Prevalence of *E. coli*

The presence of *E. coli* was confirmed in 57% of rectal swab samples using PCR, which offers higher sensitivity and specificity compared to conventional biochemical identification methods. This prevalence is comparable to findings from Brazil (70%) and Vietnam (67.3%) [23, 24], indicating that urban rodents worldwide are significant reservoirs of *E. coli*. However, the detection rate observed here was markedly higher than the 10% reported in Banjarnegara, Indonesia [25], likely due to differences in methodology, rat population density, or environmental hygiene.

### Comparative distribution of *E. coli*

No significant differences in *E. coli* prevalence were observed between species (p = 0.09) or sampling sites (p = 0.97). This suggests a relatively uniform risk of exposure across both *R. tanezumi* and *R. norvegicus*, as well as between Kampung Mandar and Lateng Villages. These areas, historically categorized as slums, are characterized by high population density, poor infrastructure, and open dumping of organic waste [26, 27, 28]. Such environmental conditions foster overlapping rat habitats and shared feeding grounds, which likely explain the similar patterns of pathogen carriage [29].

### Prevalence of Shiga toxin genes

Among *E. coli*-positive rats, 47.36% carried the *stx1* gene, while 7.01% carried the *stx2* gene. Dual-positive isolates were detected in two cases. The predominance of *stx1* is consistent with studies in other wildlife populations [30, 31]. Conversely, higher *stx2* prevalence has been reported in white-tailed deer or cattle-associated isolates [32], suggesting that species ecology and environmental exposure may influence the distribution of this gene. The greater frequency of *stx1* in this study may reflect local contamination patterns, particularly involving waste from poultry or livestock [33].

### Environmental and genetic influences

Rats in densely populated urban areas often scavenge from open garbage dumps and food markets, where decomposing meat and dairy waste may harbor toxin-producing *E. coli* [34–36]. Such conditions create a high-risk interface between rats and human-generated waste, promoting the ingestion of bacteria and interspecies microbial interactions. Horizontal gene transfer also plays a critical role, as bacteriophages, plasmids, and transposons facilitate the acquisition and persistence of virulence genes (*stx1* and *stx2*) within the rat gut microbiota [13, 37, 38]. This process may contribute to the pathogenicity of commensal strains in urban rodents.

### Public health implications

The detection of Shiga toxin-carrying *E. coli* in more than half of the wild rat population, with nearly half harboring the *stx1* gene, represents a significant public health concern. STEC strains are associated with severe human diseases, including hemorrhagic colitis and HUS [38, 39]. Urban rats, which frequently inhabit markets, food stalls, and waste sites, may serve as reservoirs and amplifiers of these pathogens [40]. Their proximity to human dwellings and food sources increases the risk of indirect zoonotic transmission through the contamination of surfaces, food, or water, particularly in informal settlements where inadequate waste management and common human–rodent contact exacerbate the risk.

### Recommendations for surveillance and control

Health authorities should incorporate rodent surveillance into broader zoonotic disease monitoring programs. Routine molecular screening of urban rat populations for pathogenic *E. coli* can act as an early warning system for public health threats [41]. Integrating rat monitoring into food safety protocols near markets and food-processing sites could help identify contamination hotspots before human cases occur. Policy interventions should emphasize community education on rodent-borne risks, proper food and waste management, and improvements in urban sanitation. Measures such as sealed waste bins, regular garbage collection, and community clean-up campaigns can reduce rodent foraging opportunities. Targeted rodent control strategies, including trapping and habitat modification, should be prioritized in high-risk zones.

### Need for a One Health approach

These findings highlight the importance of adopting a One Health approach that integrates urban environmental management with public health programs. Strong intersectoral collaboration among veterinary, health, and environmental authorities is crucial for designing sustainable strategies to prevent zoonotic diseases [42–45].

### Study limitations

This study has several limitations. First, its geographic scope was restricted to two urban villages in Banyuwangi District, limiting the generalizability of findings. Second, the short sampling period did not account for potential seasonal variation in rat populations or pathogen shedding. Third, antimicrobial resistance (AMR) profiling of *E. coli* isolates was not performed, despite rising concerns about multidrug-resistant strains in urban wildlife. Finally, the lack of parallel human or environmental sampling prevents direct epidemiological correlation between rodent-derived STEC and human health risks in the region.

### Future directions

The detection of *stx1*- and *stx2*-positive *E. coli* in wild rats underscores the necessity of continuous zoonotic disease surveillance in urban environments. Rats act as ecological sentinels, reflecting microbial contamination in areas with poor sanitation and abundant waste. The potential spillover of STEC to humans through contaminated food, informal slaughter practices, or water sources necessitates coordinated interventions involving veterinary, public health, and sanitation sectors. Future studies should incorporate AMR profiling, health assessments of captured rats, and cross-sectoral human–animal–environment monitoring to better understand transmission dynamics and guide targeted One Health interventions.

## CONCLUSION

This study provides the first molecular evidence of STEC in wild rats from urban areas of Banyuwangi District, Indonesia. Out of 100 rats captured, *E. coli* was confirmed in 57% of samples, with nearly half (47.36%) carrying the *stx1* gene, a smaller proportion (7.01%) harboring *stx2*, and 2.94% carrying both toxin genes. The predominance of *stx1* and the detection of dual-toxin-positive isolates highlight the zoonotic potential of urban rat populations, particularly in slum-adjacent environments where sanitation is poor and human–rat interactions are frequent.

The practical implications of these findings are considerable. Urban rats can act as reservoirs and amplifiers of pathogenic *E. coli*, posing risks of indirect transmission to humans through contaminated food, water, or surfaces. These results emphasize the need for municipal health authorities to integrate rodent surveillance into broader zoonotic disease monitoring programs, incorporate molecular pathogen detection into urban health surveillance, and strengthen sanitation measures, particularly in densely populated neighborhoods. Targeted rodent control, coupled with community education on hygiene and waste management, should be prioritized to reduce exposure risks.

A key strength of this study lies in its use of molecular techniques to confirm the presence of both *stx1* and *stx2* genes, ensuring higher specificity compared with traditional culture-based approaches. In addition, the study highlights a uniform distribution of infection risk across species, sex, and location, underscoring the ecological overlap of rodent populations in urban settings.

In conclusion, the detection of STEC in wild rats reinforces the importance of adopting a One Health approach that integrates veterinary, public health, and environmental management. Routine monitoring of rodent populations, coupled with urban sanitation improvements, is essential to mitigate the risks of STEC transmission. Future studies should expand geographical coverage, incorporate AMR profiling, and link rodent findings with human and environmental sampling to better understand transmission dynamics and inform effective public health interventions.

## AUTHORS’ CONTRIBUTIONS

RNP, AHW, AY, DHS, RR, and PP: Conceptualization. AHW, FLP, AY, AGRT, MAK, FR, and TA: Sampling. AHW, FLP, AY, RE, RNP, EZ, and ADFR: Sample analyses. AHW, FLP, AY, DHS, RE, RNP, EZ, and AGRT: Data analyses. AHW, AY, RE, and RNP: Writing—original draft preparation. AHW, FLP, AY, RE, RNP, TA, DHS, and MM: Writing—review and editing. AHW, RR, PP, and MM: Supervision. All authors have read and approved the final version of the manuscript.
